# Emerging role of HMGB1 in lung diseases: friend or foe

**DOI:** 10.1111/jcmm.13048

**Published:** 2016-12-31

**Authors:** Junying Ding, Xuran Cui, Qingquan Liu

**Affiliations:** ^1^ Beijing Key Lab of Basic Study on Traditional Chinese Medicine (TCM) Infectious DiseasesBeijing Research Institute of TCMBeijing Hospital of TCM affiliated to Capital Medical UniversityBeijingChina

**Keywords:** high‐mobility group box 1, receptor for advanced glycation end product, lung diseases, target therapy

## Abstract

Lung diseases remain a serious problem for public health. The immune status of the body is considered to be the main influencing factor for the progression of lung diseases. HMGB1 (high‐mobility group box 1) emerges as an important molecule of the body immune network. Accumulating data have demonstrated that HMGB1 is crucially implicated in lung diseases and acts as independent biomarker and therapeutic target for related lung diseases. This review provides an overview of updated understanding of HMGB1 structure, release styles, receptors and function. Furthermore, we discuss the potential role of HMGB1 in a variety of lung diseases. Further exploration of molecular mechanisms underlying the function of HMGB1 in lung diseases will provide novel preventive and therapeutic strategies for lung diseases.

## Introduction

High‐mobility group box 1 (HMGB1) is a highly abundant and widely expressed protein that plays a multiple role in a variety of pathological and physiological processes 1. HMGB1 has been implicated in human diseases, especially lung‐associated diseases, and is expressed in a variety of cells in the lungs such as lung epithelial cells, lung endothelial cells and alveolar macrophages 2, 3. Here, we will review the basics of HMGB1 and focus on the current understanding of connections between HMGB1 and lung diseases and its potential as a therapeutic target. This review aimed to summarize our current understanding of the role of HMGB1 in lung diseases.

## The structure and modification of HMGB1

HMGB1 is an evolutionarily conserved protein that consisted of 215 amino acids 4, 5. HMGB1 is composed of three domains: A box (amino acid residues 9–79), B box (amino acid residues 95–163) and an acidic C‐terminal tail (amino acid residues 186–215, the receptor‐binding site) 1, 6, 7, 8, 9. A box contains the antagonistic site of the B box and shows anti‐inflammatory properties *in vivo* and *in vitro*. In addition, it serves as a competitive antagonist for HMGB1 and inhibits HMGB1 activity 1, 3, 5. B box has also been identified as a function domain, which could be recognized by toll‐like receptor (TLR)‐4 10. B box and A box could bind to DNA and play a role in folding and distorting the double‐strand DNA. 89–108 amino acids of HMGB1 are responsible for binding to the receptor TLR4 11, and 150–183 amino acids are responsible for binding to receptor for advanced glycation end product (RAGE) 12. While C‐terminal tail is negatively charged, N‐terminal is composed of lysine rich in positive charge, giving HMGB1 a bipolar charge. HMGB1 contains two nuclear localization sequences (NLSs), which could stabilize chromatin structure and modulate gene transcription by bending DNA helical structure 13, 14.

**Figure 1:**
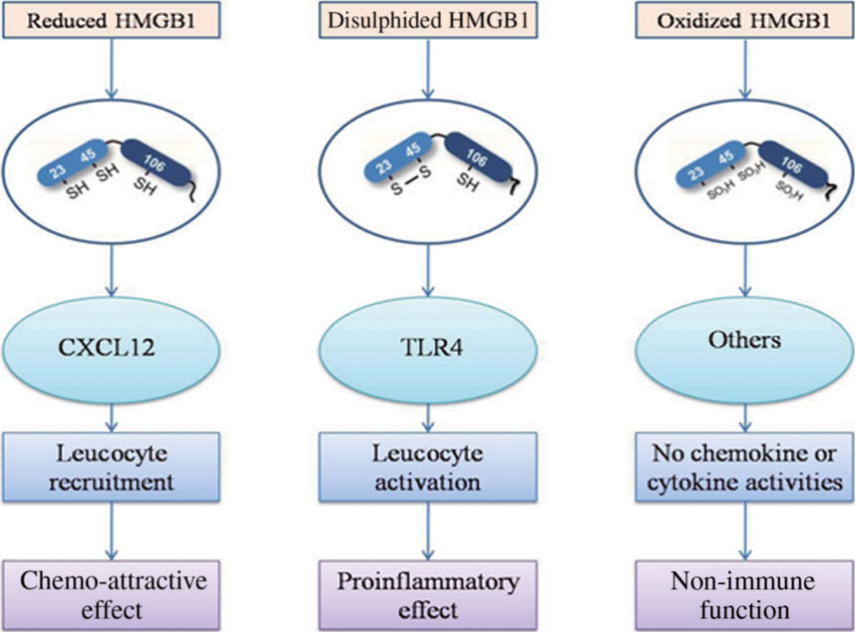
The function and molecular formula of different redox forms of HMGB1.

Multiple functions of HMGB1 largely depend on different post‐translational modifications such as acetylation, methylation, glycosylation and phosphorylation 15, 16, 17. In particular, HMGB1 acetylation is regulated by histone deacetylase (HDAC) or histone acetylase (HAT). HAT could increase HMGB1 acetylation, while HDAC could decrease HMGB1 acetylation 18. Many studies suggest the importance of redox modification in regulating HMGB1 translocation, release and activity 19, 20, 21, 22, 23. Cysteines (Cys) are modified by diverse redox signals with oxidation of side chain thiol (‐SH) to several reversible redox states such as disulphide (R‐S‐S‐R), sulfenic acid (R‐SOH) and sulfonate (R‐SO_3_H) moieties, which in turn regulate the secondary structure of proteins. Three cysteines are present within HMGB1, two vicinal cysteines in box A (C23 and C45) and a single one in box B (C106). The replacement of Cys23 or/and 45 did not affect the nuclear distribution of mutant proteins, while C106 and triple cysteine mutations could impair the nuclear localization of HMGB1, allowing the entry of some proteins into the cytosol 21. Moreover, increased endogenous and exogenous reactive oxygen species (ROS) could promote HMGB1 to translocate and release 24. The reduced C106 was necessary for the binding of HMGB1 to TLR4 and stimulating cytokine release and inflammation 25. The disulphide bond between C23 and C45 is essential for HMGB1 activity. Moreover, mutations of C45 or C23 could abolish HMGB1 cytokine activity 25 (Fig. 1).

**Figure 2:**
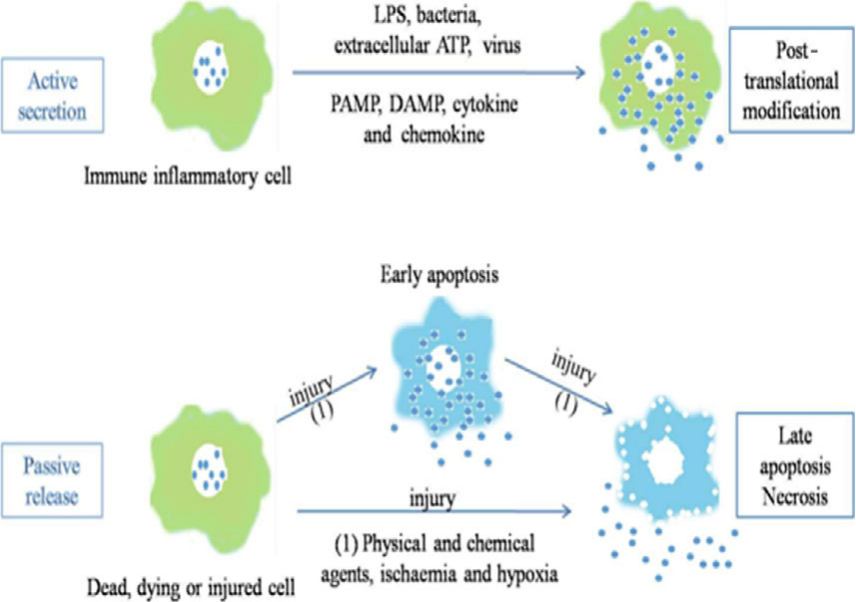
Active secretion and passive release of HMGB1.

HMGB1 could be released from active and dying cells. The different immunological and biochemical properties of HMGB1 depend on the release styles, post‐translational modifications or/and redox changes 15. The extracellular release of HMGB1 could occur by either passive or active secretion 26 (Fig. 2).

The majority of HMGB1 is usually localized in the nucleus 27. After exposure to infectious agents or endogenous danger signals, including bacteria, virus, lipopolysaccharide (LPS) and extracellular adenosine triphosphate (ATP), immune cells could mobilize nuclear HMGB1 into the cytoplasm 27, 28. When the immune cells are stimulated by external signal, some lysine residues of HMGB1 are acetylated. HMGB1 is triggered by lysophosphatidylcholine to actively secrete into extracellular matrix, and this is a crucial step in the release of HMGB1 from the activated immune cells 27.

Inflammasome participates in the regulation of LPS/ATP‐induced HMGB1 secretion 29, 30, 31. In response to danger‐associated molecular pattern (DAMP) such as ATP, pathogen‐associated molecular pattern (PAMP) such as ds‐RNA, Cp G‐DNA and endotoxin, or cytokines, immune cells, such as macrophages or monocytes, could actively secret HMGB1 in a dose‐ and time‐dependent manner 29, 32, 33, 34, 35, 36, 37, 38. Notably, extracellular ATP could induce HMGB1 release from immune cells, suggesting that immune cells could release HMGB1 actively during injury 39, 40.

On the other hand, HMGB1 could translocate during cell death and is released passively. During tissue injury, HMGB1 could be released by damaged or necrotic cells passively 41, 42. In contrast, apoptosis cells do not release HMGB1 at the early stage. During apoptosis, HMGB1 might show increased binding to chromatin, perhaps due to post‐translational modifications. As a result, HMGB1 could lose the free intra‐nuclear mobility in early stage of apoptosis, showing nuclear retention. Moreover, HMGB1 oxidation could impair its immunological activity 43, 44. In contrast, during necrosis, HMGB1 is released immediately from the cells in an active form, diffusing away for its limited chromatin binding. While it is recognized that HMGB1 location could determine the immune activity of a dead or dying cell, it is still under debate about the model of HMGB1 translocation to extracellular space during apoptosis or necrosis, which need further studies.

The status of the sulfydryl groups could explain the differences between the immune activity of HMGB1 during apoptosis or necrosis, because HMGB1 from apoptotic cells undergoes oxidation by ROS 45, 46. Following trigging to the immune cells, HMGB1 could transit from the nucleus, through the cytoplasm and into vesicles in a non‐classical secretory style 47. HMGB1 translocation occurs in immune cells or other types of cells, and post‐translational modification such as acetylation could alter the intracellular location of HMGB1 48, 49.

## HMGB1 receptor and function

**Figure 3:**
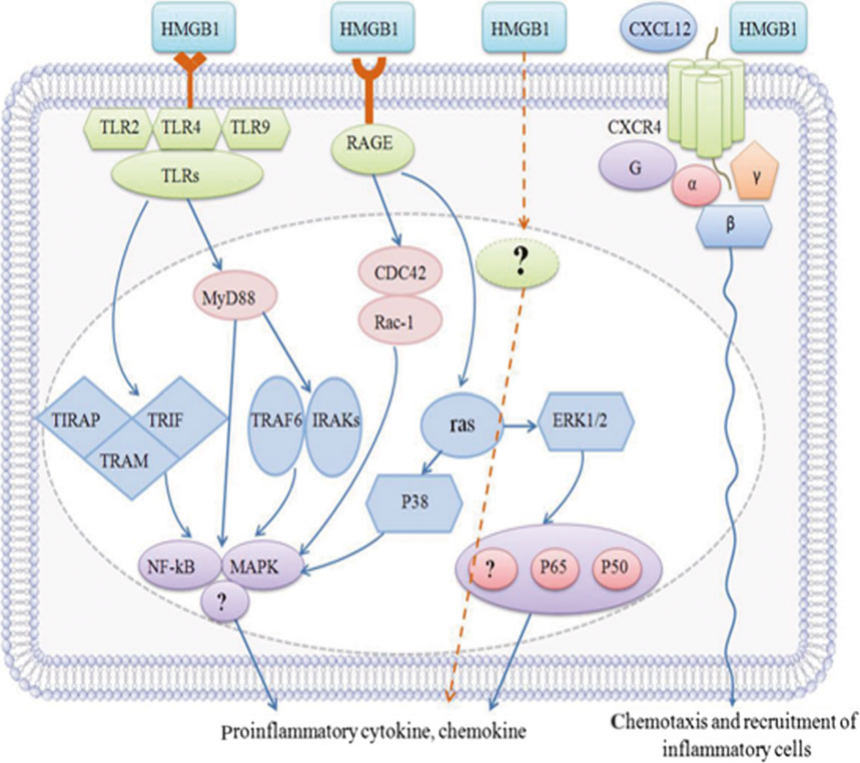
The binding of HMGB1 to the receptors and the activation of downstream signalling pathways.

HMGB1 receptors include RAGE, TLRs such as TLR2, TLR4 and TLR9, syndecan‐1 (CD138), Mac‐1, phosphacan protein‐tyrosine phosphatase (PPTP)‐ζ/β, chemokine ligand 4 (CXCL4), T cell immunoglobulin mucin‐3 (TIM‐3) and heat‐stable antigen/HAS/CD24. Among these receptors, TIM‐3 and CD24 act as negative receptors and inhibit immune activity of HMGB1 in tumour‐associated dendritic cells (TADCs) and macrophages, respectively 50, 51. Apart from direct receptor interaction, HMGB1 might form hetero complexes with other immune co‐activators such as CXCL12, nucleosome, DNA, IL‐1 or LPS that generate synergistic responses in immunity and inflammation 52. RAGE was the first receptor demonstrated to bind HMGB1 53. Apart from RAGE, HMGB1 binds TLR2 and TLR4 to induce NF‐κB activation 54. TLR4 may be more important for HMGB1‐induced macrophage activation and proinflammatory cytokine release. TLR4‐deficient animals were significantly protected from ischaemia–reperfusion injury to the kidney, liver and heart, indicating that TLR4 plays a critical role in inflammation 55. Determining the functional relationship between TLR4 and RAGE in immunity and inflammation would have important clinical significance (Fig. 3).

Once released, extracellular HMGB1 could bind to several receptors on the cell surface and activate the downstream signalling pathways such as IFN regulatory factor‐3 (IRF3), NF‐κB and phosphatidylinositol 3‐kinase (PI3K) to produce functional response 56.These receptors include RAGE, TLR, chemokine (C‐X‐C motif) receptor 4 (CXCR4), CD24 and T cell immunoglobulin mucin‐3 (TIM‐3).

RAGE is expressed on a variety of cell types and has a high affinity to HMGB1 53, 57. The binding of HMGB1 to RAGE could mediate cell proliferation, growth, migration, chemotaxis or/and differentiation and up‐regulate cell surface receptors by activating nuclear factor (NF)‐κB and mitogen‐activated protein kinase (MAPK) pathways 58, 59, 60. The cytoplasmic tail of HMGB1 is essential for intracellular signalling of RAGE, including RAGE‐mediated energy metabolism 12. In addition, RAGE provides a functional platform for the crosstalk with other receptors. For example, the interplay between TLR9 and RAGE is crucial for immune responses activated by HMGB1‐DNA complex in DC 61. The interplay between RAGE and Mac‐1 is required for HMGB1‐mediated adhesion and migration of neutrophil 62. The interplay between syndecan‐1, PPTP‐ζ/β and RAGE is required for neurite outgrowth.

TLR is also important for HMGB1 signalling pathways 63, 64. TLRs are highly conserved proteins that act as important pathogen‐recognized pattern receptors in both innate and adaptive immunity. Three members of the TLR family have been reported to be involved in HMGB1 signalling pathway: TLR2, TLR4 and TLR9, which could activate NF‐κB and MAPKs to regulate gene expression of various immune and inflammatory mediators 65, 66. TLR4‐deficient animals were protected from ischaemia–reperfusion injury to the liver, kidney and heart, which suggested that TLR4 is crucial in sterile inflammation 67. In addition, hypoxia‐induced up‐regulation of HMGB1 could promote MG‐63 cell proliferation *via* the activation of extracellular signal‐regulated kinas (ERK) and C‐Jun N‐terminal kinase (JNK) signalling in a TLR4‐dependent manner 68.

HMGB1 could promote the recruitment of inflammatory cells to damage tissue by forming a complex with chemokine ligand 12 (CXCL12) *via* chemokine receptor 4 (CXCR4) 68, 69, 70. Moreover, silencing HMGB1 promoted better resolution of keratitis caused by *Pseudomonas aeruginosa* (PA) by increasing TLRs, reducing CXCL12 and signalling through CXCR4 68. When it is released or secreted, HMGB1 could bind to CXCL12, promoting HMGB1 danger signal function 20. CD24 and TIM‐3 are negative receptors that inhibit HMGB1 immune activity in macrophages, DCs and tumour cells 71.

Necrotic cells and pyroptotic cells release the all‐thiol or completely reduced HMGB1, which could bind chemokine CXCL12 and signal through CXCR4 receptor to induce chemotaxis 69, 71. Pyroptosis caused the release of both fully reduced HMGB1 and HMGB1 with a disulphide bond in the thiol form. This form of HMGB1 could induce cytokine production by the signalling pathway *via* TLR4. Activated macrophages also release the cytokine‐inducing form of HMGB1 upon TLR4 activation. However, apoptotic cells release HMGB1 partially oxidized or completely oxidized at the critical cysteine residues. Completely oxidized HMGB1, with cysteines in the form of sulfonates, is unable to stimulate cytokines or induce chemotaxis, and apoptotic cells expressing oxidized HMGB1 could induce tolerance 72, 73.

HMGB1 passively released by apoptotic cells and HMGB1 actively secreted by activated immune cells have significant differences in molecular modifications and exhibit different function: HMGB1 released passively could induce immune tolerance, while HMGB1 secreted actively could have proinflammatory effect 74. It was found that purified recombinant HMGB1 had no inflammatory activity, but the compounds from HMGB1, ssDNA, nucleosomes and lipopolysaccharides could activate TLR family and exhibit inflammatory characteristics 75, which suggested that HMGB1 with single and complex forms plays different roles.

**Figure 4:**
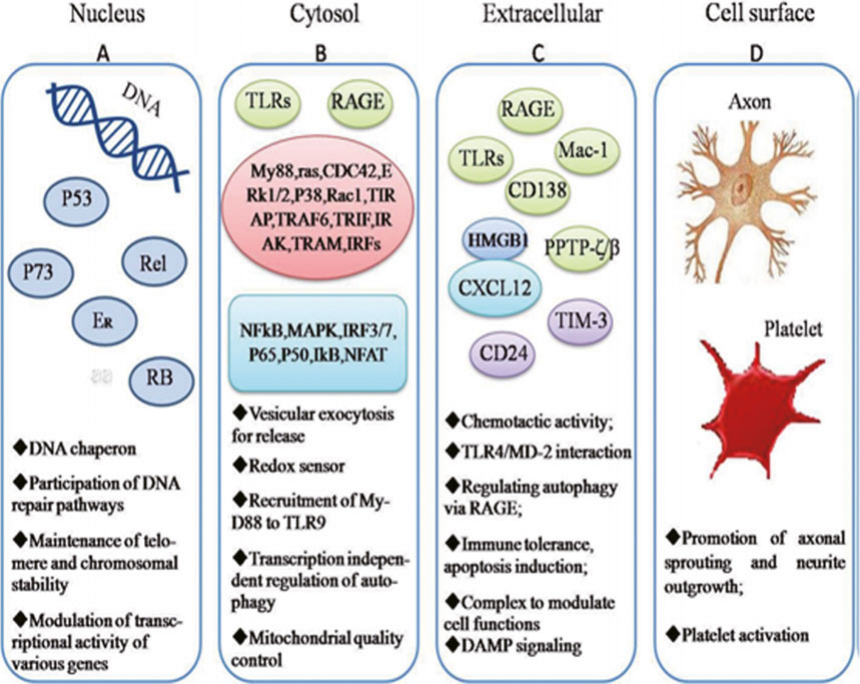
HMGB1 in different subcellular location exhibits different functions.

HMGB1 could stabilize chromatin structure and modulate gene transcription by bending DNA helical structure. The nuclear localization of HMGB1 depends on the two NLSs 47. In addition, HMGB1 could be localized to the cytoplasm, implicating that HMGB1 has important functions outside the nucleus. Acetylation of the NLSs greatly facilitates HMGB1 enrichment in the cytoplasm of immune cells or non‐immune cells 28, 76. Recent studies suggest that extracellular HMGB1 is a late mediator of sepsis and a proinflammatory cytokine 16, 36. Extracellular HMGB1 could act as a classic DAMP when released by necrotic cells, macrophages or DC in response to LPS, virus or TNF‐α 77, 78. Extracellular HMGB1 could also stimulate innate immune cells to respond to sterile injury 28, 79, eliciting an injury‐elicited systemic inflammatory response syndrome (SIRS) 4 (Fig. 4).

## HMGB1 in lung diseases

### Pneumonia

Pneumonia usually involves the inflammation of the airway end, alveolar and the lung interstitial. HMGB1 is an independent biomarker for the mortality in severe pneumonia, viral infection‐elicited pneumonia or acute respiratory distress syndrome (ARDS) (126, 131). HMGB1 is an independent biomarker for the mortality in severe pneumonia, viral infection‐elicited pneumonia or ARDS 31, 80. It was reported that HMGB1 was the best marker for discriminating between co‐infected (bacterium and virus) and single‐infected (bacterium or virus) in child bronchial pneumonia 81. HMGB1 also played a pathogenic role in hyperoxia‐induced lung injury impairment 82. Infiltrating leucocytes have the ability to secrete HMGB1 upon hypoxia, injury or inflammatory stimuli. In turn, extracellularly secreted HMGB1 could stimulate proinflammatory signalling pathway, such as the inflammasome and NF‐κB pathway, to induce the release of proinflammatory cytokines, forming a positive loop to accelerate inflammatory responses 28, 83. Furthermore, HMGB1 could be released passively from damaged cells 41. Extracellular HMGB1, as a DAMP, allowed innate immune cells to respond to the sterile injury 28, 79. Seemingly unrelated conditions such as injury and infection could converge in a common inflammation process, which is orchestrated by HMGB1 released actively or passively 31, 49, 84.

Extracellular HMGB1 could impair macrophage phagocytosis and increase the mortality of mice infected by PA 85. Moreover, 2‐O, 3‐O‐desulfated heparin (ODSH) could inhibit HMGB1 release in bronchoalveolar lavage and block neutrophil elastase (NE) stimulated HMGB1 release from murine macrophages *in vitro* 86. In addition, red blood cell (RBC) transfusion could enhance the susceptibility to lung inflammation through released HMGB1 87. Neutralizing anti‐HMGB1 monoclonal antibody conferred significant protection against PA‐induced neutrophil recruitment, bacterial infection and lung injury 88. In the presence of HMGB1, high dietary AGEs could increase RAGE expression to augment inflammatory response to aspiration 89. Taken together, HMGB1‐RAGE axis plays important role in pneumonia and is a promising target for the treatment of pneumonia.

### Pulmonary tuberculosis (PTB)

PTB is an infection disease in the lung caused by *Mycobacterium tuberculosis*. HMGB1 functions in regulating inflammation and immune response in PTB 90. In BALB/c mice infected by *Mycobacterium tuberculosis* strain H37Rv, the maximal HMGB1 concentration in bronchoalveolar lavage fluid (BALF) was at day 1 after infection, and HMGB1 is mainly from bronchial epithelium and macrophages. From day 7 to 21 after infection, the oxidized HMGB1 is predominant, while the reduced form is detected during late stage. Meanwhile, blocking HMGB1 during early infection period relieves the disease, but blocking HMGB1 activity during late infection worsens the disease 91.

In addition, plasma and sputum HMGB1 levels were significantly higher in patients with active pulmonary tuberculosis (APTB) than in healthy volunteers. Meanwhile, plasma HMGB1 levels were positively correlated with sputum HMGB1 levels and HMGB1 level was positively correlated with IL‐6 level in the plasma or sputum of patients with APTB. Moreover, HMGB1 and IL‐6 levels were positively correlated with the absolute number of monotype in patients with APTB 90.

Bacillus Calmette–Guérin (BCG) could effectively induce HMGB1 production in phorbol myristate acetate (PMA)‐treated THP‐1 cells 91. Recent studies proved that HMGB1 could act as an adjuvant for tuberculosis subunit vaccines to enhance the protective efficacy and cellular immune response, and these effects are not dependent on the interaction between HMGB1 and receptor 92. These results suggest that HMGB1 may serve as an attractive biomarker for PTB diagnosis and prognosis, and improve the efficacy of subunit vaccine as an adjuvant.

### Chronic obstructive pulmonary disease (COPD)

COPD is characterized by a progressive airflow obstruction and airway inflammation. HMGB1 is involved in the induction of airway inflammation during COPD 93. The ratio of HMGB1/soluble RAGE (sRAGE) was correlated with COPD disease status 94. Moreover, airway and systemic levels of sRAGE and HMGB1 were related to neutrophilic inflammation in COPD 95. Higher HMGB1 levels in the blood and lung were related to lung dysfunction and COPD development 96. HMGB1 could promote PASMC or PAEC cell proliferation, leading to vascular remodelling and PH pathogenesis 97. However, there was no significant correlation between plasma HMGB1 levels and longitudinal decline of lung function 98.

Recently, sRAGE was shown to contribute to airway inflammation in COPD 99. Reduced plasma sRAGE level was detected in patients with COPD, and lower plasma sRAGE level was associated with greater progression of airflow limitations in smokers with COPD 98. Taken together, these data suggest that a positive feedback loop involving HMGB1 and RAGE acts as a new driving force for airway inflammation in COPD.

Interestingly, H_2_O_2_ could induce HMGB1 translocation, expression and release in human bronchial epithelial cells 100. L. Rhamnosus and B. breve could significantly suppress cigarette smoke‐induced HMGB1 release in human THP‐1 macrophages 101. In addition, COPD airway inflammation usually involves increased inflammatory mediators such as CXCL‐8, which is a HMGB1 ligand and an important mediator for neutrophil recruitment 101.

It is known that many stressors could induce innate immune responses, inflammasome activation and inflammation with COPD. However, the mechanisms underlying pulmonary inflammation in COPD are largely unclear 102. Especially, although HMGB1 is an important mediator in multiple pathological conditions, its role in COPD has not been characterized. The correlation between the levels of HMGB1 and sRAGE or other factors in COPD has not been thoroughly clarified. Further studies on inhibiting HMGB1 and its receptor RAGE and blocking neutrophil necrosis in COPD models could provide insight into the role of HMGB1 in COPD.

### Pulmonary fibrosis (PF)

PF is a respiratory disease caused by the scars formed in the lung tissues, leading to serious breathing problems. HMGB1 levels were significantly elevated in BALs or sputum in patients with PF 88. Elevated sputum HMGB1 was correlated to 10% increased risk of lung function decline, whereas the increase in serum HMGB1 was associated with 5% increased risk of pulmonary disease progression 103. Moreover, patients with malignant pleural mesothelioma (MPM) had significantly higher HMGB1 serum levels than the population who had not developed MPM but actually had been exposed to asbestos 104. In addition, abnormal HMGB1 activation is involved in the pathogenesis of PF. In patients with PF, HMGB1 reflected the entity of pulmonary impairment and represented an independent biomarker for the progression of lung function, which could be used for monitoring lung fibrosis 103.

Inhibition of HMGB1 signalling could protect against experimental models of fibrotic diseases 105. Pulmonary rehabilitation mixture (PRM) could prevent experimental PF by modulating HMGB1/RAGE signalling pathway. PRM significantly reduced HMGB1‐mediated epithelial–mesenchymal transition (EMT) and decreased the secretion and deposition of extracellular matrix during the progression of PF. PRM inhibited TGF‐β1‐induced EMT *via* decreasing HMGB1 and increasing RAGE and E‐cadherin 106. Polymyxin (PMX) can directly absorb HMGB1 and is an attractive therapeutic option for acute exacerbation in PF 107. Collectively, these results suggest that HMGB1 is a potential therapy target for PF and the inhibition of HMGB1 by PRM, neutralizing anti‐HMGB1 monoclonal antibody or PMX, might be effective for treating PF.

### Lung transplantation

Lung transplantation was limited by chronic lung allograft dysfunction (CLAD), which may be related to macroanatomic factors, such as fragile lung parenchyma and related blood supply, and microanatomic factors, such as HMGB1 108.

Upon tissue injury, HMGB1 is released actively from the immune cells. Before and after lung transplant, increased levels of systemic HMGB1 were correlated with poor lung function, confirming the role of HMGB1 in acute lung injury (ALI) after traumatic brain injury (TBI) 109. HMGB1‐RAGE axis played an important role in brain–lung crosstalk during lung transplantation and mediated TBI‐induced lung injury in transplantation through IL‐10/IL‐17 axis 109.

A clinical transplantation study showed that increased systemic HMGB1 levels in the donors were related with impaired systemic lung oxygenation both before and after transplantation 110. HMGB1‐RAGE axis played an important role in TBI‐induced lung dysfunction, and targeting this pathway before transplant might promote recipient outcome after lung transplantation 110.

### Lung cancer

Lung cancer includes two major types: non‐small cell lung cancer and small cell lung cancer. The incidence of lung cancer is related to smoking, second‐hand smoke, exposure to toxins and family history. HMGB1 could inhibit anti‐tumour immunity, sustain inflammatory microenvironment, fulfil tumour metabolic requirements and promote angiogenesis, invasion, metastasis, genome instability and tumorigenesis.

1. *Inhibition of anti‐tumour immunity*

Cancer immunity surveillance is an important host defence process for inhibiting carcinogenesis and maintaining cellular homoeostasis. HMGB1 exhibits both immune activation and immune‐suppressive properties, depending on receptors, redox state and targeted cells 111. HMGB1 has the ability to elicit apoptosis in macrophage‐derived DCs, which could decrease host anticancer immunity 112. More recently, it was found that HMGB1 could promote tumour‐infiltrating T cells to produce lymphotoxin α1β2, leading to the recruitment of CD11b + F4/80 + macrophages into the tumour 113. In addition, HMGB1 is a chemo‐attractant for Treg, which could express HMGB1 receptors TLR4 and RAGE, and promotes the function of Treg 34. These results suggest that HMGB1 might play an important role in the anti‐tumour immunity of lung cancer.

*Sustenance of inflammatory microenvironment*

Infiltrating leucocytes could secrete HMGB1 under injury, hypoxia or inflammatory stimuli 114. In turn, secreted HMGB1 could activate proinflammatory signalling pathways. This loop could promote inflammatory responses, tumour formation and metastasis 83. It was speculated that HMGB1 plays an important role in the sustenance of lung tumour inflammatory microenvironment.

*Fulfilment of metabolic requirements of tumour*

HMGB1 has been implicated in tumour energy metabolism 115. HMGB1 in necrotic tumour cell lysates increased ATP production, providing a direct link between the inflammation and tumour energy metabolism. Extracellular HMGB1 could increase mitochondrial RAGE expression and translocation and enhance the mitochondrial HMGB1‐RAGE complex activity and ATP production 115. Loss of HMGB1 could increase mitochondrial injury and decrease ATP production. These data indicate that HMGB1 might play an important role in lung cancer progression through regulating ATP metabolism.

*Promotion of angiogenesis*

HMGB1 could bind to RAGE and activate NF‐κB pathway, inducing the expression of proangiogenic growth factors such as vascular endothelial growth factor and their receptors 116. Therefore, HMGB1 could promote the angiogenesis of lung cancer.

*Promotion of invasion and metastasis*

The inhibition of RAGE‐HMGB1 by antisense S‐oligo deoxynucleotide or the 150‐183 peptide of HMGB1 (RAGE‐binding motif) could suppress tumour cell growth, migration and invasion 12. A variety of *in vivo* and *in vitro* studies suggest that HMGB1‐RAGE signalling plays an important role in tumour invasion and metastasis. Inhibiting HMGB1‐RAGE axis is an important strategy to suppress lung cancer invasion and metastasis.

*Genome instability and tumorigenesis*

HMGB1 could modulate genome stability as HMGB1 deficiency causes genome instability 117. Loss of HMGB1 led to telomere shortening 118. The cooperation of HMGB1 and telomerase in regulating telomere length and function still remains unknown. HMGB1 could bind Topo IIα to stimulate its enzymatic activity and induce its expression, but this could be inhibited by pRb protein, suggesting that the interplay between HMGB1 and pRb could regulate Topo IIα expression and genome stability 119. HMGB1‐mediated DNA damage repair also contributes to genome stability 120. Defective autophagy is associated with genome instability, oxidative stress, inflammation and mitochondrial injury, which then contribute to tumorigenesis 121, 122. As HMGB1 is a crucial regulator of autophagy and mitophagy 123, autophagy deficiency due to loss of HMGB1 may cause genome instability and inflammation, and promote tumorigenesis of lung cancer.

## HMGB1‐targeting therapeutic agents

In the nucleus, HMGB1 plays an important role in gene expression and DNA replication. When it is released or secreted into the extracellular space, HMGB1 could act as a proinflammatory cytokine‐like mediator 124. Polyclonal or monoclonal neutralizing antibodies of HMGB1 could significantly improve survival during lethal endotoxemia or experimental sepsis 27, 28, 125. Truncation of HMGB1 into individual structural domains revealed that HMGB1 A box, a DNA‐binding motif, could specifically antagonize the activity of HMGB1 and rescue mice from lethal sepsis 126. In addition, salicylic acid (SA) could bind to HMGB1 and suppress its proinflammatory activities during sepsis 127. Neutralizing anti‐HMGB1 antibody ameliorated lung damage in a murine pneumonia model from a pathogenic strain of *S aureus* 128. In future, it will be crucial to evaluate the efficacy of HMGB1‐targeting strategies for the treatment of human sepsis 31.

HMGB1 is also involved in fibrotic disorders, especially PF. Inhibition of HMGB1 could protect against experimental models of fibrotic diseases 105. HMGB1 may play a crucial role in fibrotic diseases and thus become a promising target for treating lung fibrosis.

sRAGE or anti‐HMGB1 antibody could attenuate lung ischaemia–reperfusion (IR) damage, while recombinant HMGB1 could enhance IR injury in wide‐type mice but not in RAGE (‐/‐) mice 129. *In vitro*, after hypoxia–reoxygenation, alveolar macrophage‐derived HMGB1 could augment IL‐17 production from invariant natural killer T (iNKT) cells in a RAGE‐dependent manner 129. These results suggest that HMGB1‐mediated RAGE activation in iNKT cells is critical for the initiation of lung IR injury. HMGB1 might be the crucial intervention target of lung transplantation.

Furthermore, several HMGB1‐targeting agents have been investigated in experimental cancer models. These agents include HMGB1 neutralizing antibody, A box protein, sRAGE, ethyl pyruvate, platinating agent, quercetin, miR‐142‐3p and glycyrrhizin. Both HMGB1 neutralizing antibody and A box protein could block the activity of extracellular HMGB1 in tumour therapy 80, 130. HMGB1 release in colon carcinoma cells is mainly responsible for 5‐FU elicited leucocyte attraction and defined as a novel limiting target for combinatorial therapies, which could be prevented by HMGB1‐blocking antibodies or A box protein 131. sRAGE could act as a decoy to prevent RAGE signalling and has been used successfully in blocking HMGB1‐RAGE signalling pathway in animal tumour models 58. Ethyl pyruvate, the first HMGB1 inhibitor used in animal sepsis models to inhibit NF‐κB pathway, could inhibit tumour cell growth 132. Platinating agents had the ability to retain HMGB1 within the nucleus by conformational changes in the double helix to which HMGB1 could bind stably 133. miR‐142‐3p could down‐regulate HMGB1 and thus might be a tumour suppressor and a potential therapeutic agent for patients with NSCLC 134. The glycyrrhizin and quercetin are potential HMGB1 inhibitors and improve the effectiveness of anticancer agents 135. The beneficial and detrimental roles of DAMPs in cancer therapy have been critically evaluated, mainly focusing on HMGB1 136. An approach to individualized therapy is highly desirable in malignant disease, with circulating HMGB1 and RAGE as clinical biomarkers 137. Further investigations are required to evaluate these therapies and their promises in clinical practice.

## Conclusions and perspectives

Despite significant progress in our understanding of HMGB1 structure, release styles and receptors, intracellular signalling pathways downstream of HMGB1 remain relatively poorly defined, especially determining the functional relationship between TLRs and RAGE would be of great interest.

Large human patient populations with well‐defined clinical lung diseases and animal lung disease models are required to systematically characterize the role of HMGB1 in corresponding lung diseases. In addition, although HMGB1‐specific antagonists have been shown to be effective in animal models of diverse lung diseases, further preclinical and clinical investigations are required to evaluate their potential application in clinical practice.

Interestingly, traditional Chinese medicines have shown potential as HMGB1‐targeting therapies. Glycyrrhizin (GZA) could bind to HMGB1 and inhibit its secretion or action. Epigallocatechin‐3‐gallate (EGCG) could stimulate autophagic HMGB1 degradation. Tanshinone IIA could stimulate endocytic HMGB1 uptake. Zhimu could regulate HMGB1 signalling and treat diabetic peripheral neuropathy. Moreover, herbal extracts, such as Danggui, Mungbean and Prunella Vulgarisnicotine, and active components, such as nicotine, EGCG, tanshinone, glycyrrhizin, chlorogenic acid, rosmarinic acid, isorhamnetin‐3‐O‐galactoside, persicarin, forsythoside B, chloroquine, acteroside and shikonin, are effective in inhibiting endotoxin‐induced HMGB1 secretion 138.

In conclusion, in this review, we summarize recent advances on the understanding of the role of HMGB1 in lung diseases. It is obvious that different disease conditions lead to the abnormality of HMGB1 expression, modification, release or secretion, which then triggers the activation of signalling pathways that regulate inflammatory response. Therefore, targeted pharmacological interventions against HMGB1 using its antibodies, inhibitors or antagonists, inflammatory inhibitors and even traditional Chinese medicines would become novel therapeutic approaches for lung diseases.

## Conflict of interest

The authors declare no conflict of interest.
